# Evolution of Job Satisfaction and Burnout Levels of Emergency Department Professionals during a Period of Economic Recession

**DOI:** 10.3390/ijerph17030921

**Published:** 2020-02-02

**Authors:** Aurora Fontova-Almató, Rosa Suñer-Soler, Laia Salleras-Duran, Carme Bertran-Noguer, Laura Congost-Devesa, Marta Ferrer-Padrosa, Dolors Juvinyà-Canal

**Affiliations:** 1 Department of Nursing, Faculty of Nursing, University of Girona, 17003 Catalonia, Spain; aurora.fontova@udg.edu (A.F.-A.); laia.salleras@udg.edu (L.S.-D.); carme.bertran@udg.edu (C.B.-N.); dolors.juvinya@udg.edu (D.J.-C.); 2 Emergency Department, Empordà Health Foundation, Figueres, 17600 Catalonia, Spain; lcongost@salutemporda.cat (L.C.-D.); mferrer@salutemporda.cat (M.F.-P.)

**Keywords:** economic recession, job satisfaction, burnout, professional, emergency service, hospital, health personnel, nursing, health promotion

## Abstract

Satisfaction at work has been found to be a predictive factor of permanency. On the other hand, burnout has been associated with financial loss. The purpose of this study was to analyse the levels of satisfaction and burnout of professionals in a hospital emergency department and make a comparison with results from the same service during the economic recession in 2012. An analytical, cross-sectional and descriptive study was undertaken during two time periods into the levels of satisfaction and burnout of the professionals of an emergency department. Consequently, 146 replies were received. The percentage of professionals who considered their salary to be unsatisfactory in 2012 diminished in comparison with 2018 (*p* = 0.034), while job stability was considered more satisfactory in 2018 (*p* = 0.039) and the timetable in 2018 as more unsatisfactory (*p* = 0.009). With regards to burnout, it was observed that in 2018 the score for depersonalisation had fallen (*p* = 0.029) in comparison with 2012. An improvement in the level of satisfaction is observed in 2018, and more positive scores have also been found in the depersonalisation subscale in 2018. An inverse association was observed between depersonalisation in 2018 and overall satisfaction.

## 1. Introduction

Europe’s recent economic recession has led to structural changes in health services [1]. Many European countries have been subject to austerity measures as a result of the economic crisis, which have added pressure to health systems [2]. A report of the World Health Organisation (WHO) analysing the economic crisis and health between 2007 and 2013 claims that cutting the cost of health professionals has been the price that has been paid for making savings in healthcare [3]. In the case of Spain, both the number of professionals and the salaries received by them were reduced as a direct response to this crisis [3]. Working conditions and pay have been considered as important factors in the retention of professionals in their positions, in maintaining their motivation and in incentivising improvements in terms of their productivity and performance [3]. 

Job satisfaction, which encompasses the individual feeling that people have with regards to their work and an evaluation of the extent to which their professional needs are fulfilled [4], is a predictive factor of permanency for both doctors and nurses [5,6]. It has been observed that a satisfied worker feels greater engagement with their work [7].

Taking the theory of Maslow, some investigators approached job satisfaction from the perspective of the satisfaction of needs [8]. However, the focus has now shifted largely to cognitive processes [9]. In contrast to the traditional vision, Herzberg and Mausner [10] postulated that satisfaction and dissatisfaction were two separate phenomena, which could even be considered as having no relationship between one another. They considered that intrinsic factors, which they called “motivators” (factors related with the work that is done) gave satisfaction and that extrinsic factors, which they called “hygiene factors”, resulted in dissatisfaction. Herzberg and Mausner’s motivator-hygiene theory has dominated the study of the nature of job satisfaction and constituted one of the theoretical bases for the development of the evaluation of job satisfaction. It has been observed that both intrinsic rewards (the satisfaction of work well done, a challenging job, a sense of achievement) and extrinsic rewards (salary, stability and job security) have an influence on the job satisfaction of qualified professionals [7]. Earlier studies affirm that intrinsic factors determine job satisfaction whereas extrinsic factors determine dissatisfaction [11,12]. Satisfaction questionnaires of professionals have been considered an indicator of the quality of service of an emergency department [13]. Measuring the quality of the health services, which can be done by controlling indicators that permit monitoring, enables continuous improvement [14]. Furthermore, the characteristics of the working environment have been clearly associated with patients’ results in several studies, especially with regards to their safety and satisfaction [15,16,17].

In contrast, professional burnout syndrome, was studied as a response to workplace stress, affecting the health of the personnel as well as their professional and social relationships [18]. The syndrome is characterized by high levels of emotional exhaustion, depersonalization and a feeling of low personal accomplishment and has diverse individual and environmental causes [19,20]. Burnout has recently been defined as a work-related condition consisting of exhaustion, loss of control over emotional and cognitive processes, and mental distancing [21]. Pressure at work can lead workers to a situation of burnout and this can lead to financial loss related to absenteeism, changes in the position of work, and health problems [22].

Job satisfaction and burnout syndrome have been well-studied in health professionals in hospital settings. However, there has been less research into job satisfaction in emergency services [23,24]. A connection has been established between burnout syndrome and job satisfaction as an independent variable, while other sociodemographic variables can also have an influence [25]. In a meta-analysis of 31 studies of satisfaction in nursing professionals, job stress had the strongest negative correlation with job satisfaction [26]. 

More recently, it has been reported that nurses and physicians reported greater stress and work pressure than administrative staff and described a worse working environment. Specifically, it has been seen that job satisfaction among nurses and physicians in emergency departments is lower than that of administrative staff, with the former perceiving greater stress and work pressure [27]. 

Other researchers have observed a significant relationship between burnout and job satisfaction, and emotional exhaustion, specifically, is found to be a significant predictor of all three dimensions of job satisfaction while depersonalisation was not significant [24].

Given this background, the present investigation aims to analyse the levels of satisfaction and burnout of professionals in a hospital emergency department and make a comparison with results from the same service during the economic recession in 2012, when healthcare workers suffered from a significant loss of income and lack of career progression, and 2018, by which time the staff had their previous conditions restored to them, taking as a hypothesis that levels of job satisfaction and levels of burnout of the professionals of an emergency department are related to one another, and that they are influenced by the economic situation being experienced. 

The study contributes to further our knowledge in two ways: (1) to advance in the scientific knowledge regarding the relationship between job satisfaction and burnout syndrome in professionals of a hospital emergency service; (2) to discover how workers are affected by occupational changes in periods of economic crisis.

## 2. Material and Methods

Analytical, cross-sectional, and descriptive study (two time periods) of satisfaction, motivation and professional burnout of the professionals of the emergency department of a country hospital through the use of questionnaires from October to December 2012 and November 2017 to January 2018. 

Professionals working during the months of the study were included (doctors, nurses, secretarial staff, nursing assistants and porters) and those professionals who were on leave during the months of data collection were excluded.

### 2.1. Variables Studied

*Sociodemographic variables:* age, sex, civil status (married or with a partner, single, widowed, divorced), and number of children (open field). The civil status was grouped into two categories for later analysis (married or with a partner and others).

*Occupational variables:* professional category (doctor, nurse, nursing assistant, secretary, porter), occupational status (permanent or temporary contract), shift worked (mornings, afternoons, nights, substitutions), length of service in the emergency department (in years), and job satisfaction (on a scale from 0 to 10, with 10 being the highest possible score). 

*Other variables:* having responded to the survey of 2012 (yes/no), the general satisfaction scale and the Maslach burnout inventory.

*Overall Job Satisfaction Scale*: validated in Spanish [12,28] consists of 15 items, each one of which allows 7 different responses (extremely dissatisfied, dissatisfied, moderately dissatisfied, unsure, moderately satisfied, very satisfied, and extremely satisfied), assigning a value from 1 to 7, which we have recoded for analysis as three items. Only one answer can be chosen for each one of the items. From the sum of these responses, three scores are obtained corresponding to overall satisfaction, intrinsic satisfaction and extrinsic satisfaction. Overall satisfaction, which is obtained from the sum of all of the items, can oscillate between 15 and 105 points, with a higher score reflecting greater overall satisfaction. Intrinsic satisfaction is composed of the 7 items and scores can oscillate between 7 and 49. Extrinsic satisfaction consists of 8 items and scores oscillate between 8 and 56 [11,12]. 

*Maslach Burnout Inventory (MBI):* consists of 22 items divided into three dimensions, which are evaluated with a Likert-type scale. This instrument has been validated for use with health professionals [29].

The emotional exhaustion dimension (*EE*) is made up of 9 items that make reference to the reduction or loss of emotional resources or describe feelings of saturation or emotional exhaustion due to work; the dimension of depersonalisation (*D*) is made up of 5 items that describe a cold and impersonal response and lack of feelings and insensitivity towards those receiving attention; and the personal accomplishment in work dimension (*PA*) consists of 8 items that describe feelings of competence and success at work. In this last dimension, low scores are indicative of burnout [29].

### 2.2. Collection of Data and Procedure

Data were gathered through a questionnaire that we created containing the previously described variables. Three points were set up in the emergency department where a letter providing information to the professional, informed consent forms, a data collection booklet, and a return box were made available. An email was sent to the professionals on starting the collection of data and one week before the end of the period.

Professionals were informed by email that the questionnaires would be distributed and of the objectives of the study. Three different points were established to deposit questionnaires and it was not possible to associate responses with the names of professionals. Only the principal investigator had access to the questionnaires, which carried a numeric code. 

### 2.3. Ethical Considerations

This project respects the Helsinki Declaration of the World Medical Association on the ethical principles for medical investigations into humans. The ethical principles with regards to research set out in Organic Law 3/2018 regarding the Protection of Personal. The confidentiality of the professionals has been maintained at all times. The research project was approved by the research committee of the centre and by the hospital’s Ethics and Clinical Investigation Committee.

### 2.4. Statistical Analysis

Data was recorded in two Microsoft Excel^®^ V16.33 for Mac (Madrid, Spain) spreadsheets to maintain anonymity and was analysed using the IBM SPSS Statistics^®^ V19 software package (Madrid, Spain).

Numerical variables were described with the mean and standard deviation or the median and interquartile range. Categorical variables were described in frequencies and/or percentages. The chi-squared test was used for categorical variables and the non-parametric Mann-Whitney U test was used to compare quantitative and categorical variables. 

A single unic lineal regression model was made to study the factors associated with the satisfaction of professionals, adjusted by age and the dimensions of depersonalisation and personal accomplishment of the MBI. Significance was taken as *p* < 0.05. A comparative analysis of the results obtained from the same questionnaire in 2012 was performed. 

## 3. Results

### 3.1. Patient Characterisation

A total of 146 questionnaires were received: 81 (55.5%) in 2012 and 65 (44.5%) in 2018. Further, 51.7% of the people surveyed people in 2018 had responded to the questionnaire conducted in 2012. The response rate was 71% in 2012 and 71.4% in 2018.

The mean age in 2018 was 40.1 years (SD = 9.9) and 73.8% of respondents were women. The sociodemographic and occupational variables of the people who responded to the questionnaire in 2012 and 2018 are given in Table 1.

### 3.2. Job Satisfaction

The median score for job satisfaction of the professionals in both 2012 and 2018 was 7 (IQR = 2). 

In the case of the overall satisfaction scale each item was first analysed separately, as is set out in Table 2. It was observed that the percentage of professionals that considered their salaries to be unsatisfactory had reduced in 2018, *p* = 0.034 (chi-squared test), the timetable in 2018 was considered more unsatisfactory than in 2012, *p* = 0.009 (chi-squared test), and that job stability was considered as being more satisfactory in 2018, *p* = 0.039 (chi-squared test).

The results of the scores of the overall satisfaction scale in 2018 were a median of 68.0 in overall satisfaction (IQR = 20.5), 34.0 in intrinsic satisfaction (IQR = 10.5), and 35.0 in extrinsic satisfaction (IQR = 7.75). No differences were observed between these scores and those recorded in 2012 (Mann-Whitney U test), as can be seen in Table 3.

### 3.3. Maslach Burnout Inventory

The burnout scores of the workers in 2012 and 2018 were compared. It was observed that the score in the depersonalisation subscale had fallen, *p* = 0.029 (Mann-Whitney U test), and that there was a tendency to improvement in personal accomplishment, as can be observed in Table 4.

The sociodemographic and occupational variables were compared with job satisfaction and the Maslach burnout inventory in 2012 and 2018. In 2012 it was observed that nurses scored higher in intrinsic satisfaction than the rest of professionals. On the other hand, in 2018 doctors scored higher than the rest of professionals in overall, intrinsic and extrinsic satisfaction. Emotional exhaustion was positively related to age in 2012 and with years of service in 2018. Personal accomplishment was inversely related with years of service. Table 5 shows the results of the analysis. 

### 3.4. Regression Model

In order to study the factors strongly associated with satisfaction, an overall satisfaction multivariate model was created to examine the interactions between variables that were shown to be associated in the bivariate analyses from the data gathered in 2012 and 2018. An inverse association was observed between depersonalisation in 2018 and overall satisfaction, as can be seeing Table 6. Neither age nor the personal accomplishment dimension were associated with the overall satisfaction of the workers. 

## 4. Discussion

In the present study, data gathered in 2012 and 2018 were compared. More than half of the people surveyed said that they had responded to the two questionnaires. In a study on burnout in a local government in England at two time periods to observe the effects of economic recession [30], a response rate of 54% was obtained for a second questionnaire conducted 12 months after the first. The mean age of the participants and the number of children had increased in the sample of 2018 with respect to 2012. On the other hand, in this study by Ravalier et al. [30] no significant differences were observed in the age and sex of the participants between the two time periods, but these were found with regards to length of service. 

In the present study, the global scores for overall, intrinsic and extrinsic satisfaction did not differ between the samples of the different years. On comparing the items of the satisfaction scale separately between 2012 and 2018, it was observed that satisfaction had improved with regards to salary and job stability but had worsened with regards to the timetable. These items are extrinsic factors that do not generate satisfaction [11,12], which may explain why there were no differences in the overall score. 

On making a comparison with other studies, it is observed that in a comparative study between doctors in Iceland and Norway, the Icelandic doctors scored low with regards to satisfaction with their salaries, and lower in satisfaction than the Norwegians [1]. It has been shown in earlier studies that stress, leadership and salary are associated with job satisfaction and of nurses giving up their jobs [4]. Furthermore, in some investigations, it has been observed that the factors that increase job satisfaction among doctors are the variety of the work, the relationships and contact with colleagues, and teaching medical students. On the other hand, the factors that reduce satisfaction with work are salary, the hours worked, the workload, the lack of time, and lack of recognition [31]. In a study conducted with doctors in Iceland, it was observed that those who had low levels of job satisfaction had a greater intention of leaving to work abroad [6].

In the present study, the scores for satisfaction and burnout have been studied with reference to sociodemographic variables, with it being observed that the intrinsic satisfaction of nurses was higher in 2012. In 2018, the overall, intrinsic, and extrinsic satisfaction of the doctors was higher than the other professional categories, which is a finding that differs from the results of Suarez et al. [27]. 

With regards to burnout syndrome, it was observed that the score for depersonalisation was lower in the sample of 2018. On the other hand, the investigation of Ravalier et al. [30] observed that emotional exhaustion and depersonalisation increased in the second survey, but that personal accomplishment also increased, although the data analysed were from 2012. Our data from 2018 give similar scores to those obtained in a study undertaken of emergency doctors and nurses in a province of Catalonia in 2017 [32], although the score in the professional accomplishment dimension was higher than that of our study. 

Jones et al. [33] defined the concept of stressors related with recession as workplace stressors such as the increase in occupational workloads or the reorganisation of tasks as a result of the economic crisis. These stressors are positively associated with pressure at work and negatively associated with satisfaction. Pressure is associated with a reduction in job satisfaction, increased absenteeism, counterproductive behaviour and turnover [33]. The stressors that showed a greater association with pressure were the reorganisation of the work, increased workload, and training restrictions. On the other hand, the results that most affected satisfaction were delays in being paid, restrictions on training and reorganisation of the work. In our study, satisfaction in relation to job stability and the salary had improved although it had reduced with regards to the timetable, a fact which could explain the improvement in the burnout dimensions.

With regards to the regression models, an inverse relationship was observed between depersonalisation and overall, intrinsic and extrinsic satisfaction in 2018, whereas this relationship was not observed in the data from 2012. This can be explained by observation of the bivariate analysis in which it is seen that some satisfaction variables as well as depersonalisation had improved by 2018. This is in line with earlier studies [34] in which an association between burnout and satisfaction was observed. 

The influence of the economic crisis on the satisfaction of the professionals has not been directly measured, but professional burnout and satisfaction have been measured. In the satisfaction scale, we have been able to observe how satisfaction and dissatisfaction have changed with regards to certain aspects, such as salary, timetable, and stability of employment, which may be due to organisational changes to adapt to the economic recession. Maresso et al. [35] describe restrictions in benefits such as holidays and sick leave, increases in the weekly timetable, the failure to substitute staff on leave or following retirement, and a cut of 7.14% in the salaries of professionals in Spain in 2012 coinciding with the data from the first survey. 

Our hypothesis regarding the association between job satisfaction and burnout has been partially supported by the results, given that job satisfaction is found to have a strong inverse association with the depersonalisation dimension of burnout, but not with the other two dimensions. 

### 4.1. Limitations

The results of the present investigation need to be interpreted with caution as the study has been conducted at a single hospital, and only in an emergency department, which may not reflect the conditions in other areas of the hospital. 

Although the response rate was high, due to staff turnover and other reasons only half of those who were surveyed in 2018 had responded to the earlier survey. 

### 4.2. Implications in Clinical Practice

Satisfaction with work and professional burnout are factors that influence the health of professionals and have economic effects on healthcare sector companies. This research enables us to see how measures applied during a period of economic recession have affected the levels of satisfaction and burnout of workers and, where these effects have been negative, control of the determining causes of changes in the satisfaction of professionals may avoid the application of these measures in the futures. Additionally, knowledge of the items that generate the most satisfaction may help in the implementation of strategies to improve satisfaction and reduce burnout among professionals. 

Future lines of investigation should study how healthcare organisations have adapted to the economic recession and how this has affected both the professionals working in the health systems and users. 

## 5. Conclusions

An improvement has been observed in satisfaction between 2012 and 2018, specifically with regards to salary and job stability. On the other hand, satisfaction with regards to the timetable decreased.

With regards to the dimensions of burnout, more positive scores were found in the subscale of depersonalisation in 2018 than in 2012. However, there were no significant changes in the dimensions of personal accomplishment and emotional exhaustion.

Finally, in the multivariate model, an inverse association was observed between depersonalisation and overall satisfaction in 2018.

## Figures and Tables

**Table 1:** Sociodemographic and occupational variables of the emergency service professionals (*n* = 146).

Variable	*n* (%) 2012	*n* (%) 2018	*p*
**Age (mean, SD)**	35.9 (9.4)	40.1 (9.9)	* 0.027
**Sex**			** 0.707
Men	19 (23.5)	17 (26.2)	
Women	62 (76.5)	48 (73.8)	
**Civil status**			** 0.230
Married/with partner	56 (69.1)	50 (78.1)	
Single	22 (27.2)	11 (17.2)	
Divorced	3 (3.7)	3 (4.7)	
**Number of children**	median 0 (IQR = 1)	median 1 (IQR = 2)	* 0.038
Without children	34 (55.7)	25 (38.5)	
1 child	13 (21.3)	15 (23.1)	
2 children	12 (19.7)	22 (33.8)	
3 children	2 (3.3)	2 (4.6)	
**Professional category**			** 0.871
Doctor	23 (28.4)	18 (27.7)	
Nurse	36 (44.5)	33 (50.8)	
Nursing assistant	7 (8.6)	6 (9.2)	
Secretarial staff	10 (12.3)	5 (7.7)	
Porter	5 (6.2)	3 (4.6)	
**Shift worked**			** 0.888
Mornings	13 (16.7)	9 (13.8)	
Afternoons	12 (15.3)	8 (12.3)	
Nights	7 (9.0)	7 (10.8)	
Alternating shifts	46 (59.0)	41 (63.1)	
**Type of contract**			** 0.883
Permanent	64 (79.0)	52 (80.0)	
Temporary	17 (21)	13 (20)	
**Years of service in emergency department**	median 9 (9.5)	median 12 (14)	* 0.103

Tests used: * Mann-Whitney U test, ** Chi-squared test.

**Table 2:** Results of the comparison of items of the overall satisfaction scale in 2012 and 2018.

Scale Items		Total% *n* = 139	2012% *n* = 78	2018% *n* = 61	χ^2^; *p*
**Physical conditions**	Dissatisfied	24.8	20.0	30.8	2.89; 0.235
	Unsure	23.4	22.5	24.6	
	Satisfied	51.7	57.5	44.6	
	Total	100	100	100	
**Freedom to choose work method**	Dissatisfied	9.0	6.2	12.5	1.96; 0.375
	Unsure	24.1	23.5	25	
	Satisfied	66.9	70.4	62.5	
	Total	100	100	100	
**Work colleagues**	Dissatisfied	5.5	8.6	1.6	3.47; 0.177
	Unsure	4.8	4.9	4.7	
	Satisfied	89.7	86.4	93.8	
	Total	100	100	100	
**Recognition**	Dissatisfied	32.4	34.6	29.7	0.39; 0.823
	Unsure	25.5	24.7	26.6	
	Satisfied	42.1	40.7	43.8	
	Total	100	100	100	
**Immediate supervisor**	Dissatisfied	8.9	9.9	7.7	0.26; 0.877
	Unsure	14.4	14.8	13.8	
	Satisfied	76.7	75.3	78.5	
	Total	100	100	100	
**Assigned responsibility**	Dissatisfied	6.2	4.9	7.8	0.64; 0.727
	Unsure	22.1	23.5	20.3	
	Satisfied	71.7	71.6	71.9	
	Total	100	100	100	
**Salary**	Dissatisfied	56.2	60.5	50.8	6.77; 0.034
	Unsure	17.1	21.0	12.3	
	Satisfied	26.7	18.5	36.9	
	Total	100	100	100	
**Possibility of using skills**	Dissatisfied	12.3	16.0	7.7	2.34; 0.310
	Unsure	16.4	16.0	16.9	
	Satisfactory	71.2	67.9	75.4	
	Total	100	100	100	
**Relationship with the management**	Dissatisfied	63.0	67.9	56.9	2.16; 0.339
	Unsure	18.5	14.8	23.1	
	Satisfied	18.5	17.3	20.0	
	Total	100	100	100	
**Possibilities of promotion**	Dissatisfied	52.4	56.3	47.7	1.41; 0.494
	Unsure	25.5	25.0	26.2	
	Satisfied	22.1	18.8	26.2	
	Total	100	100	100	
**Management of the service**	Dissatisfied	28.8	25.9	32.3	2.09; 0.351
	Unsure	24.0	28.4	18.5	
	Satisfied	47.3	45.7	49.2	
	Total	100	100	100	
**Attention to suggestions**	Dissatisfied	25.3	25.9	24.6	0.099; 0.952
	Unsure	25.3	25.9	24.6	
	Satisfied	49.3	48.1	50.8	
	Total	100	100	100	
**Timetable**	Unsatisfactory	30.3	20.0	43.1	9.36; 0.009
	Unsure	16.6	17.5	15.4	
	Satisfied	53.1	62.5	41.5	
	Total	100	100	100	
**Task variety**	Dissatisfied	8.9	11.1	6.2	1.57; 0.456
	Unsure	15.8	13.6	18.5	
	Satisfied	75.3	75.3	75.4	
	Total	100	100	100	
**Job stability**	Dissatisfied	24.7	29.6	18.5	6.49; 0.039
	Unsure	11.0	14.8	6.2	
	Satisfied	64.4	55.6	75.4	
	Total	100	100	100	

Note: Tests used: Chi-squared test.

**Table 3:** Comparison of the scores of the overall satisfaction scale of the years 2012 and 2018.

Overall Satisfaction Scale	2012	2018	*p*
**Overall**	[78] 65.8 (11.1); 68 (15)	[61] 68.3 (12.5); 68 (20.5)	0.236
**Intrinsic satisfaction**	[78] 31.3 (5.6); 31.5 (7.5)	[61] 32.5 (6.6); 34 (10.5)	0.254
**Extrinsic satisfaction**	[78] 34.5 (6.6); 35 (9.2)	[61] 35.8 (6.9); 35 (8.5)	0.545

Note: Test: Mann-Whitney U test. The score of the dimensions is described with [n], mean score (SD) and median (IQR).

**Table 4:** Comparison of Maslach Burnout Inventory scores in 2012 and 2018.

Maslach Burnout Inventory	2012	2018	*p*
Emotional exhaustion	[81] 18.8 (10.1); 16 (14)	[61] 18.7 (9.4); 18 (15)	0.737
Personal accomplishment	[81] 33.8 (8.6); 35 (12)	[61] 36.6 (6.6); 37 (10)	0.052
Depersonalisation	[81] 8.1 (5.5); 8 (7)	[61] 6.1 (4.9); 6 (6)	0.029

Note: The score of the dimensions is described with [n], mean score (SD) and median (IQR).

**Table 5:** Relationship of satisfaction and the Maslach Burnout Inventory in 2012 and 2018 with sociodemographic and occupational variables.

Variables	Overall Job Satisfaction Scale 2012			Overall Job Satisfaction Scale 2018			Maslach Burnout Inventory 2012			Maslach Burnout Inventory 2018		
	Overall	Intrinsic	Extrinsic	Overall	Intrinsic	Extrinsic	EE	PA	D	EE	PA	D
Age * (ρ; *p*)	ρ = −0.147; *p* = 0.475	ρ = −0.118; *p* = 0.398	ρ = −0.080; *p* = 0.570	ρ = 0.079; *p* = 0.546	ρ = 0.007; *p* = 0.959	ρ = 0.203; *p* = 0.107	ρ = 0.368; *p* = 0.007	ρ = −0.010; *p* = 0.943	ρ = −0.146; *p* = 0.296	ρ = 0.217; *p* = 0.090	ρ = −0.128; *p* = 0.316	ρ = 0.048; *p* = 0.708
Sex **	*p* = 0.152	*p* = 0.003	*p* = 0.886	*p* = 0.231	*p* = 0.142	*p* = 0.630	*p* = 0.357	*p* = 0.030	*p* = 0.386	*p* = 0.516	*p* = 0.868	*p* = 0.923
Men	62 (11.8); 64 (18)	27.8 (5.2); 29 (8)	34.3 (7.2); 35 (10)	64.5 (11.2); 64 (14)	30.1 (7.3); 29 (9)	34.4 (5); 35.5 (4.2)	17.5 (10.7); 13 (15)	29.8 (9.9); 30 (14)	9.2 (6); 8 (12);	17.6 (8.6); 14 (16)	36.2 (7.4); 39.5 (10)	7.1 (7.3); 3 (12)
Women	67.0 (10.7); 69 (15)	32.5 (5.2); 33 (9)	34.7 (6.5); 35 (9.7)	69.5 (12.8); 69.5 (24)	33.3 (6.2); 34 (10.5)	36.1 (7.4); 35 (12.2)	19.2 (10); 16 (12)	35 (7.8); 36 (9)	7.7 (5.3); 7 (6);	19.2 (9.7); 18.5 (15);	36.8 (6.2); 36 (9)	5.8 (4); 6 (6)
Civil Status **	*p* = 0.708	*p* = 0.408	*p* = 0.856	*p* = 0.911	*p* = 0.731	*p* = 0.529	*p* = 0.980	*p* = 0.346	*p* = 0.685	*p* = 0.830	*p* = 0.961	*p* = 0.330
Married/with partner	65.5 (11.7); 68 (15.5)	31 (6); 31 (6)	34.7 (6.8); 35 (9)	68.1 (12.2); 68 (15)	32.6 (6.7); 34 (10)	35.4 (6.5); 35 (6.5)	19.2 (11); 15.5 (15)	34.2 (9); 37 (12)	8.5 (6.2); 7.5 (11)	18.8 (9.8); 17 (17)	36.8 (6.3); 38 (9)	6.4 (5.2); 6 (8)
Other	66.4 (9.8); 67 (13.7);	32.3 (4.5); 32 (8);	34.4 (6.3); 35 (11);	68.9 (13.6); 66.5 (26.5);	32 (6.3); 30.5 (11.5);	36.9 (8.1); 38 (17);	17.8 (8); 16 (13);	32.8 (7.5); 35 (14);	7.1 (3.3); 8 (5);	8.7 (8.3);18.5 (14);	36.5 (7.4); 39 (12);	4.9 (3.6); 5 (5);
Professional category ***	*p* = 0.101	*p* = 0.002	*p* = 0.848	*p* = 0.012	*p* = 0.014	*p* = 0.011	*p* = 0.527	*p* = 0.664	*p* = 0.122	*p* = 0.907	*p* = 0.369	*p* = 0.628
Doctor	69.2 (12.8); 65.5 (18.7)	29.7 (6.1); 30 (7.7)	33.6 (7.3); 35 (10)	73.8 (13.6); 77.5 (13)	34.7 (7); 36 (9)	39.2 (7.2); 40 (9)	19.6 (8.4); 20 (13)	34.6 (6.3); 35 (10)	7.2 (5.1); 7 (6)	18.9 (8.6); 20.5 (15)	36 (6.4); 36.5 (8)	5.7 (5.5); 4.5 (5)
Nurse	68.8 (10.3); 72 (14)	33.7 (5); 33.5 (7.7)	35.3 (6); 36 (9)	67.7 (11.3); 68 (14)	33 (5.7); 32 (10)	34.5 (6.3); 34 (8.5)	18.1 (10.5); 15 (12)	34.8 (7.6); 35.5 (11)	7.3 (5.1); 7 (7)	19.4 (10.8); 18 (19)	36.2 (6.7); 34.5 (11)	6.5 (5.1); 6 (8)
Others	63.7 (9.6); 63 (13.5);	29.2 (4.4); 29 (5.5);	34.7 (6.9); 35 (11.5)	61.4 (10.7); 59.4 (10.5)	28 (6.2); 26.5 (10)	33.7 (6.3); 34 (8)	19 (11.5); 14 (14);	31.3 (11.5); 34 (17)	10.1 (6.0); 9 (8)	17.4 (6.4); 16 (9)	38.7 (6); 41 (6)	5.7 (3.5); 6.5 (6)
Shift worked ***	*p* = 0.517	*p* = 0.898	*p* = 0.178	*p* = 0.201	*p* = 0.303	*p* = 0.243	*p* = 0.376	*p* = 0.462	*p* = 0.743	*p* = 0.225	*p* = 0.381	*p* = 0.486
Mornings	66.1 (15.2); 63 (23)	31 (7); 29 (10)	35.1 (8.7); 35 (12.5)	77.8 (13.3); 84 (25)	35.7 (6.2); 38 (9)	41.2 (7.4); 41 (14.7)	22.3 (13.4); 19 (25)	31.6 (10.1); 32 (11)	8 (5.8); 7 (10)	25 (12.2); 26 (18)	33.7 (8.8); 32 (17)	6.1 (5.1); 5 (6)
Afternoons	70.1 (8.6); 72 (12)	32.6 (5.5); 33 (9.5)	37.6 (4.5); 38 (7)	69.4 (13.8); 68 (27)	33.1 (5.8); 34 (10)	36.4 (8.1); 35.5 (15.2)	16.2 (8); 14.5 (12)	35 (10.5); 37.5 (14)	7 (5.3); 5.5 (10)	15.7 (10.5); 14 (22)	38.63 (8.3); 41.5 (11)	5.2 (3.8); 6.5 (7)
Nights	66.3 (9); 69.5 (6.7)	30.8 (5.5); 33 (7)	36,8 (6); 38 (6.7)	64.8 (7.1); 66.5 (13.5)	28.7 (5.7); 29 (8)	34.8 (5.3); 37 (11)	14.3 (7.9); 14 (7)	36.9 (6.1); 36 (12)	6.7 (1.2); 7 (3)	21.5 (9.8); 22.5 (15)	35.5 (4.1); 34 (8)	8.8 (4.9); 8.5 (8)
Alternating Shifts	64.9 (10.7); 65.5 (13.5)	31.4 (5.3); 31.5 (8);	33.4 (6.3); 33.5 (9.2)	66.9 (12.3); 68 (19.5)	32.4 (6.7); 34 (10.5)	34.5 (6.4); 35 (9)	19.5 (9.9); 17 (14)	33 (7.8); 34.5 (13)	8.6 (5.7); 8 (8)	17.9 (8.2); 16 (14)	37.1 (5.8); 38 (9)	4.3 (5.1); 6 (7)
Type of contract **	*p* = 0.114	*p* = 0.073	*p* = 0.451	*p* = 0.311	*p* = 0.150	*p* = 0.821	*p* = 0.044	*p* = 0.120	*p* = 0.593	*p* = 0.134	*p* = 0.205	*p* = 0.186
Permanent	65 (10.8); 65 (14.5)	30.8 (5.5); 31 (7)	34.5 (6.4); 35 (10)	67.5 (12.9); 68 (19.5)	31.8 (6.7); 32 (9.7)	35.5 (7); 35 (7)	20 (10.4); 17.5 (15);	33 (9.1); 33 (13)	8 (5.6); 7 (7)	19.7 (9.1); 20 (15)	36.2 (6.5); 36 (10)	6.5 (4.8); 6 (7)
Temporary	68.5 (12.1); 71 (16)	33.3 (5.4); 35 (7)	35.2 (7.5); 37 (9)	71.1 (10.7); 73 (20.5)	34.9 (5.7); 36 (9.5)	36.2 (6.3); 35 (9.5)	14.3 (7.5); 13 (6)	36.8 (5.3); 38 (6)	8.3 (4.9); 8 (8)	16 (10.5); 14 (13)	38.4 (6.5); 41 (9)	4.8 (5.2); 4 (7)
Years of service in emergency department * (ρ; *p*)	ρ = 0.008; *p* = 0.952	ρ = −0.055; *p* = 0.659	ρ = 0.112; *p* = 0.369	ρ = −0.001; *p* = 0.993	ρ = −0.083; *p* = 0.507	ρ = 0.140; *p* = 0.275	ρ = 0.238; *p* = 0.051	ρ = −0.172; *p* = 0.161	ρ = 0.018; *p* = 0.884	ρ = 0.288; *p* = 0.024	ρ = −0.287; *p* = 0.024	ρ = 0.131; *p* = 0.312

Note: Test Used: * ρ = Rho de Spearman, ** Mann-Whitney U test, *** Kruskal-Wallis test. The score of the dimensions is described with mean score (SD) and median (IQR); *p* < 0.05 EE: Emotional exhaustion PA: Personal accomplishment; D: Depersonalisation.

**Table 6:** Regression models for overall satisfaction in 2012 and 2018.

	Dependent Variable: Overall Satisfaction				
	B	SE	CI 95%	β	*p*
Year 2018	−9.677	15.424	−39.909 to 20.555	−0.419	0.532
Age 2012	−0.113	0.158	−0.424 to 0.197	−0.187	0.476
Age 2018	0.241	0.150	−0.053 to 0.536	0.431	0.111
Depersonalisation 2012	0.004	0.293	−0.572 to 0.580	0.002	0.988
Depersonalisation 2018	−1.057	0.331	−1.706 to −0.408	−0.411	0.002
Personal accomplishment 2012	0.176	0.180	−0.177 to 0.529	0.268	0.331
Personal accomplishment 2018	0.332	0.250	−0.158 to 0.823	0.547	0.187
Multiple R^2^	0.21				

Note: B: coefficient B; SE: standard error; CI 95%: confidence interval of 95%; β: standardized beta coefficient. Multiple R^2^: R-square, the coefficient of determination.
